# Shoulder-to-shoulder: healthcare professionals’ experiences of learning during electronic health record implementation in municipal primary care

**DOI:** 10.1080/02813432.2026.2673131

**Published:** 2026-05-16

**Authors:** Christin Uvsløkk Wiggen, Kirsti Sarheim Anthun, Erna Håland, Monica Lillefjell

**Affiliations:** ^a^ Department of Neuromedicine and Movement Science, Norwegian University of Science and Technology (NTNU), Trondheim, Norway; ^b^ SINTEF Digital, Health Research, Trondheim, Norway; ^c^ Department of Education and Lifelong Learning, Norwegian University of Science and Technology (NTNU), Trondheim, Norway

**Keywords:** Electronic health records, primary care, workplace learning, implementation, communities of practice

## Abstract

**Background:**

Formal training is commonly used in electronic health record (EHR) implementation, but often fails to address healthcare professionals’ ongoing, context-specific learning needs. There is limited knowledge of how learning unfolds in practice when such training is experienced as insufficient, particularly in municipal healthcare services.

**Methods:**

This qualitative study examined learning processes during the implementation of a new EHR system in a municipal healthcare institution in Central Norway. Data was generated through interviews and observation, and was analyzed thematically.

**Results:**

Participants experienced formal training as poorly aligned with their digital competencies and everyday clinical work. Limited time and staffing further constrained participation in formal training and follow-up. Alongside formal training, employees engaged in peer-based learning practices embedded in everyday work, described as ‘shoulder-to-shoulder’ learning. These practices became particularly important due to limited time and staffing, enabling rapid problem-solving and mutual support, while also reinforcing shared responsibility for learning. Rather than merely compensating for inadequate training, shoulder-to-shoulder learning emerged as an important adaptive practice that supported problem-solving and collaboration during EHR implementation.

**Conclusion:**

Learning to use EHR systems in municipal primary care should be understood as a socially embedded and ongoing process. While formal training may provide initial orientation, implementation strategies should also recognize and support peer-based learning practices that support ongoing learning and collaboration during periods of organizational change.

## Introduction

Over the past decades, the digitalization of the healthcare sector has received increasing attention. International organizations such as the World Health Organization and the OECD point to a rapid expansion in the use of information and communication technologies, which affects how healthcare services are delivered across different contexts [1,2]. A core of this development is the adoption of health information technology, particularly Electronic Health Records (EHRs). According to the World Health Organization’s Global Strategy on Digital Health, EHRs are central in supporting the development of more coordinated, patient-centered care [2]. OECD analyses similarly highlight the potential of EHR systems to enhance patient safety, improve the quality of care, and support more efficient use of healthcare resources [1].

Despite the many documented benefits of EHRs, such large-scale digital systems often entail significant challenges, especially related to employee training and adapting the technology to existing work procedures and local workflows [3,4]. In settings or services with limited staffing, there is less time for tailored support and training, which makes it more challenging for employees to adapt the system to their own routines and areas of responsibility [5,6].

### EHR implementation in a Nordic and Norwegian context

Similar concerns have been reported in Nordic studies, indicating that although EHR training programs are designed to be comprehensive, they fail to address the practical tasks that clinical work consists of [7]. Studies illustrate that healthcare professionals meet challenges related to changes in work procedures and the training quality, which affects perceived usefulness and the ability and motivation to master EHR system use [7,8].

In Norway, the structure of the healthcare system has created unique challenges for integrating EHR systems. The Norwegian health system is organized into municipal health services and regional health authorities. The municipalities are responsible for primary healthcare, and the regional health authorities are responsible for specialist healthcare [9]. As a result of this, municipalities and hospitals have previously used different health record systems. This has led to fragmented information flow and communication challenges between the services [10].

With the aim of meeting these challenges, a new EHR system, the Health Platform (HP), has been implemented in Central Norway [11]. The Health Platform is based on Epic technology [12]. Although such systems are often portrayed as well-integrated and comprehensive, international research highlights several challenges associated with their implementation. These often involve user-friendliness, increased workload and documentation burden, as well as a lack of alignment with clinical workflows [3,13,14]. Evaluations of the HP implementation in its early stages have reported similar concerns, particularly regarding patient safety and the strain on everyday clinical work [15,16].

These developments illustrate that the introduction of large-scale EHR systems entails substantial changes in everyday work practices, which in turn place new demands on how healthcare professionals learn to use and integrate such systems in practice.

### The need for practical and continuous learning in EHR implementation

Empirical studies provide insight into how employees experience learning processes during EHR implementation. Poor usability and insufficient local support have been shown to undermine users’ confidence and motivation, thus also affecting the effectiveness of learning to use the system [17,18]. Other studies emphasize the importance of support from colleagues and superusers in developing employees’ digital skills [19,20], and that nurses in primary care call for more targeted training to integrate technology into their daily work [21,22].

Several studies also point to the value of practical, role-specific, and experience-based training, as well as blended learning, and suggest that these types of training promote learning [22–24]. Findings from EHR implementation research also suggest that training cannot be limited to a single session before system roll-out. Instead, learning requires ongoing organizational support, sufficient time for training, and opportunities for staff to share knowledge and experiences [25,26].

### Experiences with training and learning in EHR implementation

Evaluations of the Health Platform in Central Norway emphasize that despite the training program being both structured and based on evidence-based recommendations, many employees wanted more practical and experience-based training [27]. Follow-up studies suggest that EHR training rarely reflects actual use in everyday clinical work. In response, employees often develop informal support networks and local adaptations to manage the system in practice [28–30]. This alignment between the training and actual practice may also lead to ineffective system use, frustration and reduced motivation among healthcare professionals, which can result in a lack of preparedness to use the EHR system in everyday practice [31,32]. These experiences illustrate that formal training with limited adaptation to local work practices is insufficient on its own to meet the need for continuous, context-sensitive learning in practice. While such informal and peer-based learning strategies are highlighted as valuable resources in these contexts, previous research has noted that this could lead to fragmented learning, workarounds, and potential implications for patient safety [33,34].

In the context of EHR implementation, a distinction is often made between training and learning. Training often refers to structured, course-based activities designed to introduce users to system functionalities [35]. Learning, however, often also unfolds outside formal activities as healthcare professionals engage with real work tasks, collaborate with colleagues, and adapt the technology to their local workflows [13]. Understanding how such workplace-based learning processes develop during implementation may therefore provide important insight into how healthcare professionals manage technological change.

### Research gaps and study purpose

Previous research has highlighted the importance of peer support and informal learning when implementing EHR systems [21,22], while less attention has been paid to how such learning processes take place in a municipal healthcare setting with limited training resources and access to personnel [4,5]. This indicates a need to investigate more closely how healthcare professionals learn to use EHR systems in practice and how they compensate for insufficient training activities. Against this background, we ask: How do healthcare professionals experience and engage in training and learning processes during the implementation of an EHR system in a municipal healthcare setting?

### Theoretical framework: a sociocultural perspective on learning

To deepen our understanding of how healthcare professionals experience and participate in learning processes during the implementation of an EHR system, this study builds on a sociocultural learning perspective [36]. This perspective assumes that learning does not primarily take place individually, but also through social participation within communities of practice. It is in concrete situations, through language, collaboration, and joint problem solving, that knowledge is developed and given meaning. Vygotsky [36] stated that learning occurs through the active use of language in social contexts. To understand how learning is supported through interaction with others, the concept of Zone of Proximal Development (ZPD) becomes relevant. Vygotsky uses this concept to describe how people learn most effectively when they receive support to perform tasks that are just beyond what they can master without support, but which become understandable through receiving guidance from others. Later, Wood et al. [37] built on these ideas and introduced the concept of scaffolding, which is a metaphor to illustrate how more experienced colleagues or mentors help learners develop understanding and skills. Learning thus appears to be situated, closely linked to the specific context and to the relationships and practices that characterize the work community.

Furthermore, our study builds on the theory of Communities of Practice (CoP), as developed by Lave and Wenger [38], and further elaborated by Wenger [39]. A CoP is developed when people work together towards a joint aim, interest, or field of expertise. Over time, they develop a common language, routines, and shared understandings of what the work entails. Learning occurs through participating in the community and by being a part of an environment where knowledge is shared and developed through practice. Wenger [39] further describes CoPs as being characterized by mutual engagement, shared repertoire, and joint enterprise. Joint enterprise represents a collective understanding of what the community aims for and how they work to sustain this purpose through practice. Mutual engagement and shared repertoire capture how individuals interact and how they develop a set of collective tools, languages, and understandings. These shared ways to understand, speak, and act may take the form of locally adapted routines, common terminology, templates or workflows, and other artifacts that gradually become ‘how we do things here’ within a community [38,39].

### Study setting – The implementation of the Health Platform in Central Norway

The study was conducted at a municipal nursing home that provides both long-term and short-term care services, located within a health and care center in a medium-sized municipality in Central Norway, as classified by Statistics Norway [40]. The municipality shares characteristics typical of other Norwegian municipalities, such as limited staffing levels, varying availability of expertise, tight budgets, and relatively frequent staff turnover [41]. In settings or services with limited staffing, there is less time for tailored support and training, which makes it more challenging for employees to adapt the system to their own routines and areas of responsibility [5,6].

The study was conducted in the context of the implementation of the Health Platform (HP). The system was implemented at the municipal health and care center in early 2023 as part of the regional rollout of the Health Platform. The system was developed to standardize and improve the flow of information, as well as to streamline documentation and collaboration across health services. The implementation brought about significant changes in employees’ documentation practices and workflows. Employees received support through training programs and support mechanisms throughout the implementation phase. This included training tailored to users’ professional roles prior to the rollout, digital training materials, and access to local superusers who provided support during the early stages of system use. The training procedure offered to healthcare staff followed a predefined training strategy combined with local adaptation. The training procedure was developed by Helseplattformen AS, a publicly owned company responsible for the procurement, implementation, and management of the Health Platform electronic records system in Central Norway.

The training was organized into three phases: a maturity phase, a system-learning phase, and a skills-training phase. As reported in an evaluation report of the training program [27], employees were prepared for the change through information meetings and digital resources prior to the roll-out of the EHR system. The main goal was to build an understanding of why the new system was introduced and what it would mean in practice. Formal training included e-learning courses on topics such as information security, system navigation, login, and access. In addition, classroom sessions were tailored to specific roles, such as commercial staff, nurses, and doctors. These sessions were structured around standardized workflows that reflected typical work tasks.

After roll-out, learning was meant to occur through daily use of the system, with support from colleagues and local superusers, and with less focus on formal training activities. Superusers were part of the implementation strategy and served as local resources during the EHR system introduction. They were ordinary employees who received extended training in the system, and their task was to support colleagues in the daily use of the system through facilitation close to the practice field and problem-solving. Superusers did not have formal leadership responsibility but provided important local support after the system was put into use [27].

## Materials and methods

### Research design

This study employs a qualitative research design, with data collected through individual semi-structured interviews and one semi-structured group interview. Semi-structured interviews were chosen as they are well-suited to explore participants’ experiences in relation to complex practices, while allowing flexibility to follow issues raised by the participants themselves [42]. To gain contextual insight into the work setting, a short observation period was conducted in addition to the interviews. The study was guided by an interpretive approach [43,44].

### Sample and recruitment

A total of 12 participants participated in the study (10 nurses, 1 department manager, and 1 implementation coordinator). Eight participants participated in individual semi-structured interviews, including six nurses, the department manager, and the implementation coordinator. Two nurses were interviewed together in a group format, and two nurses were observed for six hours while performing daily tasks.

All participants were employed within the same municipality. All but one were employed at the same healthcare institution; one participant held a municipal administrative role related to the system introduction. All participants used the EHR system in their daily work during the implementation period, and some also had roles related to the local implementation. Participants’ ages ranged from approximately 35–60 years, and their professional experience in the healthcare sector ranged from about 10–40 years. The participants thus represented a range of ages and expertise.

A contact person at the institution facilitated recruitment by distributing invitations to relevant participants. A strategic sampling approach [45] was used to target staff who used the EHR system in their daily work.

### Data collection

We collected data through semi-structured interviews using an interview guide with open-ended questions covering the introduction and integration of the EHR, learning experiences, and related challenges. The interview guide used across all phases covered the same topics, with adjustments made to allow participants to reflect on experiences gained over time. The guide was also adapted to participants’ professional roles. While the overall themes remained the same, some questions were adjusted or omitted in the interviews with the department manager and the EHR coordinator to reflect their respective responsibilities during the implementation process.

The EHR system was implemented in the municipality in early 2023. Data collection took place across different points throughout the implementation period. The six-hour observation of everyday work practices and training activities, and interviews with the implementation coordinator and the department manager were conducted in Spring 2023, shortly after the system’s introduction. Individual interviews with nurses were conducted in summer 2023, when participants had gained more experience using the system in their daily work. The group interview was conducted in fall 2024, after the system had been in use for a longer period and after several adjustments had been made to both the system and local work practices had taken place. The participants interviewed in these phases were different individuals, and each was interviewed only once to capture situated perspectives from healthcare professionals involved in the implementation, which together formed a single dataset exploring patterns in their learning experiences during EHR implementation.

All interviews, except for one, were conducted at the participants’ workplaces during their working hours and lasted about 40–60 min. One individual interview was conducted digitally via Zoom due to practical reasons. The first author conducted all interviews. The individual interviews were conducted together with the second author, while the group interview was conducted by the first author. One interview was conducted in a group format because the participants collaborated closely in their daily work and shared experiences related to the implementation process, which allowed them to reflect collectively on these experiences. While the group format enabled participants to build on each other’s experiences, it may also have influenced the responses, as participants could shape or reinforce each other’s perspectives during the discussion. All interviews were recorded with participants’ consent and transcribed verbatim by the first author shortly after the interviews were conducted.

In addition, a six-hour observation session was conducted shortly after the rollout of the EHR system. The observation was made by the first author, who followed two nurses who also had roles as superusers. The observation was conducted during their daily work tasks and focused on how challenges with the EHR system were met and resolved. Although the observation was limited in scope, it provided contextual insight into everyday work practices related to the use of the EHR system [46].

### Researcher reflexivity

The first author conducted all interviews and the observation. The first author’s background in sociology and workplace learning informed the study’s focus, particularly on how learning occurs in collaboration with colleagues and through concrete work practices. The individual interviews were carried out together with the second author, whose expertise includes healthcare, organization, digitalization, and social anthropology. The remaining authors contributed perspectives from health services research and sociology, including public health, implementation processes, learning, and organizational development. Together, these complementary perspectives informed discussions of the material and guided the development of the analytical interpretations.

None of the authors have been employed by the organization behind the journal system, nor have we had roles as consultants or advisors in its implementation. This position outside the implementation work may have made it easier for the participants to speak openly about their experiences, while at the same time, it has been necessary to reflect on how our professional interest in implementation and learning could influence both the data collection and the interpretation of the material [47].

To support this work, a reflective log was kept during data collection, in which methodological choices, impressions from interview situations, and preliminary analytical reflections were documented on an ongoing basis. Interpretations and the development of themes were then discussed in the research group. Such reflective involvement on the part of the researchers is central to reflexive thematic analysis [48,49].

### Method of analysis

We conducted the analysis following Braun and Clarke’s [48] six-phase approach to reflexive thematic analysis and began with repeated readings of the transcribed material and observation notes. We chose this approach because it enables the identification of patterns of meaning across heterogeneous qualitative material and allows both explicit and more interpretive patterns to be explored.

All interviews were audio recorded and transcribed verbatim by the first author. Participants were assigned anonymous identifiers during the transcription process. The first author conducted the initial coding inductively using Microsoft Excel. The first author identified text segments relevant to the research question and assigned preliminary codes in an initial coding spreadsheet. Both interview transcripts and observation notes were coded in this phase. While the interviews constituted the primary analytical material, the observation notes mainly provided contextual understanding of work practices and supported the interpretation of participants’ accounts. The first author then grouped the codes into broader patterns of meaning in a separate spreadsheet. Through an iterative process of reviewing and grouping related codes, the first author gradually developed these patterns into candidate themes.

While the first author conducted the primary coding and initial theme development, the emerging interpretations and thematic structure were discussed with the research team. The co-authors contributed by questioning interpretations and helping refine the developing themes.

The analysis focused on themes addressing the aim of this article: to explore how healthcare professionals experience and engage in learning processes during the implementation of an EHR system. The themes presented in this article, therefore, capture learning processes at the micro level of everyday work practices. The coding process was informed by a sociocultural perspective on learning, which views learning as embedded in activity, interaction, and context [39]. This perspective informed the interpretation of codes and the development of themes, which is in line with reflexive thematic analysis [48]. Table 1 presents examples of initial codes contributing to each theme and sub-theme.

**Table 1.** Overview of main themes, subthemes, and example initial codes

Main themes	Subthemes	Example initial codes
Lack of relevance and adaptation in formal learning activities	Insufficient context-based content	Overwhelming training; Too much information in a short time; Limited practice-oriented training; Training without real patient cases
	Lack of adaptation to digital skills and learning needs	Lack of profession-specific training; One-size-fits-all training; Individual learning needs; Age-related differences in learning
Time limits and lack of resources as barriers to learning	Time pressure and conflicting priorities	Lack of time for training; Time as a bottleneck for learning; Time pressure in everyday work; Lack of room for reflection
Informal and peer-based learning as a compensatory strategy	‘Shoulder-to-shoulder’ learning in everyday work	Shoulder-to-shoulder learning; Observation followed by practice; Informal guidance in daily work; One-to-one guidance as a safe learning space
	Collective learning responsibility and mutual support	Collective responsibility for learning; Collegial support; Low threshold for asking for help; Trust-based collegial environment; Informal knowledge sharing

### Ethical considerations

This study is a part of a larger project, funded by the Norwegian Research Council (NFR) (Project number: 326988), which aims to explore the relationship between digitalization, training, and professional practice in municipal health care services. The study adheres to the ethical guidelines established by the Norwegian Research Ethics Committees (NESH).

All participants were informed about the study’s purpose and procedures and provided written informed consent, including details on confidentiality, anonymization, and data storage. They were also informed of their right to withdraw from the study at any time without consequences. All data were securely stored and anonymized.

The study was reviewed by the Norwegian Agency for Shared Services in Education and Research (SIKT) and was found to meet national ethical standards for research (protocol code: 659487).

## Results

The findings are presented in three main themes that describe how healthcare professionals experience and engage in learning processes during the implementation of the EHR system. Each main theme is accompanied by sub-themes, which are organized under their respective themes following the coding and categorizing process.

### Main theme 1: Lack of relevance and adaptation in formal learning activities

Many participants found the formal training course held before rollout overwhelming, too far removed from their actual practice, and ineffective for their learning. They expressed frustration over the volume of information delivered within a short timeframe, leading several to feel frightened or demotivated afterwards. They also pointed out that the training did not take into account the participants’ actual work tasks or the differences in their digital competence, which resulted in decreased motivation and poorer learning outcomes.

#### Sub-theme 1: Insufficient context-based content

Participants reported several challenges with the predefined training measures, particularly the session held several months prior to rollout. This training took place in a large sports hall, where each participant had an individual desk equipped with two PC screens, and the instructor provided one-way instructions. The session lasted eight hours, during which the instructor demonstrated how to navigate the system. Participants found the content poorly aligned with their actual work tasks, leaving them feeling confused and overwhelmed rather than learning something useful.

We had the training session at *anonymized place*, maybe too early … We felt that it was less relevant because we didn’t yet understand how the system would work in practice. There was too much information packed into one day. And then we thought ‘it will be ok when we get our own patients’ because it did not become practice-based for us. (Informant 2)

We learned a lot unnecessarily. It became just chaos, really. We could have … When we started with Profile (our previous EHR system), we began very simply, and then we learned very simple things. We also had a new training session where we took it a step further. It should have been a bit more like that, I think. Because it was … it was a whole day, you know, and it was huge (the EHR system). I almost felt like it slammed into my head in the end, complete chaos. (Informant 1)

These quotations illustrate participants’ experiences of being overwhelmed by the complexity of the EHR system and their perception that the training did not support gradual learning. The lack of breaks and time to reflect further compounded the issue.

#### Sub-theme 2: Lack of adaptation to digital skills and learning needs

Another finding was that participants felt the training did not sufficiently accommodate differences in their digital skills. While some felt that they had mastered the system, others felt demotivated and frightened because their first encounter with the system made them question their own capacity to learn it.

I think maybe a lot of people were a bit scared. Because I think about … at least those who are a generation older than us, they don’t have that kind of digital vocabulary. They don’t have a clue what scrolling is, you know. So there’s something about learning things like that. In addition, we’re going to have a telephone, a PC, and yes … ‘Where do I start’. So I think they experienced it as chaotic and that they were a bit scared. (Informant 7)

As we can see from this quote, certain actions, such as ‘scrolling’, may seem unfamiliar to some, and the combination of having to deal with phones, PCs, and new, unfamiliar digital workflows creates a sense of chaos in the first encounter with the system. This illustrates how differences in digital experience influenced how participants perceived the training and their ability to engage with the new system.

### Main theme 2: Time constraints and staffing shortages as barriers to learning

Participants describe a clear conflict of priorities between taking time for training and participating in superuser meetings, as those assigned to this role are expected to do. A common view among participants is that patient care always comes first, leaving little time to prioritize training. One participant, who also holds the role of superuser, describes how clinical patient responsibility always takes precedence over training activities, including attending formal meetings.

I think the understanding is there, but the resources and money and stuff, I think that’s where it stops. Because we should have had a few more people at work. We would probably have had more time to work with the health platform then. I am called to a number of meetings as a superuser; we are a cluster and part of a professional cluster, and we also have superuser meetings. However, it is very rare that I have time, as I prioritize patients first, because they are the reason I am here. I can’t leave my colleagues if they can’t administer medication, or if they are temporary or unskilled. […] And so I have declined these meetings several times because I haven’t been able to attend, due to a lack of resources in the ward. (Informant 2)

This example illustrates how patient responsibilities and limited staffing levels took priority over planned training activities. When superusers need to prioritize patient needs over attending meetings, this disrupts both their own and their colleagues’ competence development. The absence from superuser meetings not only leads individuals to miss out on information about the journal system but also limits the superuser’s opportunity to share information and guidance with colleagues. These experiences with superusers’ absence from formal meetings also mirror those of other employees, who find that the general training is becoming scarce. As another participant says:

But then there’s the issue of finding the time and opportunity to practice a little during a normal working day. That’s the biggest frustration… We don’t have time to sit and practice; we don’t have time to sit and get to know each other. I know I should have learned this, and I’m embarrassed that I still don’t know how to do it. […] It looks so easy when you do it, but then it’s probably three weeks before we have another opportunity to practice, and by then I’ve forgotten how to do it. I should have kept at it. (Informant 8).

Although arrangements are made for employees to practice, staffing constraints often leave insufficient time and space to step away from clinical work and practice using the new EHR system. The lack of repetition, ongoing practice, and opportunities to consolidate what they had learned made it difficult for participants to maintain confidence in using the system, and knowledge of the system remained uncertain and fragmented.

### Main theme 3: Informal and peer-based learning as a compensatory strategy

The participants’ descriptions reflect a strong working community that takes collective responsibility for learning. Despite experiencing insufficient training that is not context-related, and the lack of time and resources, this does not prevent employees from engaging in the learning process. Through collective learning processes *in situ*, which participants often refer to as ‘shoulder-to-shoulder’ learning, they solve challenges and problems on an ongoing basis in situations that arise during the working day. Participants described a strong sense of shared responsibility for helping each other learn how to use the EHR system.

#### Sub-theme 1: ‘shoulder-to-shoulder’ learning in everyday work

‘Shoulder-to-shoulder’ practice reflects spontaneous knowledge sharing that arises *in situ*. When questions and uncertainties arise about the EHR system during patient care situations or in the duty room in between tasks, they pause for a moment, explain the steps, and continue with the task. Such short, practical sequences, done shoulder-to-shoulder, link learning directly to actual tasks, which helps to increase relevance and reduce the memory loss between the course day and the usage situation.

We help each other. It’s kind of an expression, shoulder-to-shoulder. […] It’ informal, we do it while we work. It can take one minute, and it can take five. Now I’ve just been asked ‘how do I introduce medicine again’, ‘so you do this and that’, and I’ve shown her. It’s in the duty room in between doing other tasks, it’s quick, there’s nothing formal … like taking out of the department to have meetings. (Informant 4)

#### Sub-theme 2: Collective learning responsibility and mutual support

Employees make knowledge sharing an integral part of their working day by taking the few minutes needed to solve a specific problem. This means that learning takes place precisely when one encounters a challenge, and that a colleague who already masters the technical procedure immediately steps in as a mentor:

The advantage of shoulder-to-shoulder is that we are able to help colleagues become better at something, and that … We have time, but when it happens it might be … it might be five minutes or ten minutes, but it’s the fact that we get to increase the competence of the person who needs help, and that the person who has already learned it can contribute to learning it faster. […] If you haven’t attended training, you’ll still be able to learn, because someone else knows it, so we just have to learn from each other. (Informant 6)

Two patterns appear in these descriptions: One is rapid response to real needs, which anchors new knowledge in the task at hand. The other is that the role of both participants in the learning situation strengthens the individual’s ownership of the learning process: when they teach others, they also learn more in-depth themselves. The work community develops a competence base; even if some have not participated in training, they still have the opportunity to learn, as knowledge is shared within the community.

The same impulse and responsibility to fill colleagues’ knowledge gaps is also evident in the following description:

If I know something, I teach it to my colleague. That’s what we’ve done. […] I’ve done that most recently today, when I see a colleague sitting and writing an evaluation using the old method, and now we’re going to do it in a new way, I said ‘I’ll show you how to do it afterwards’, and we teach each other. Because then I have that knowledge, and I share it with her. She also knows how to do something else: ‘Yes, but now I’ve seen that if you do this and that, you’ll get in there’ on a flow chart … and ‘Yes, but I want to see how you do that, can you show me’. So that’s what I put into this, the shoulder-to-shoulder concept, and then we do it. Informal internal teaching. (Informant 2)

Challenges related to system use are often coped with directly when they arise, based on shared experiences and solutions between colleagues. Through such informal exchanges, small, practical learning moments arise as spontaneous contributions in the collegial community. Employees take responsibility for supporting each other, and this mutual sharing contributes to competence development, even when formal training is perceived as inadequate.

Importantly, these situated learning processes are deeply embedded in an existing workplace culture characterized by close collaboration. Several participants described ‘shoulder to shoulder’ as a familiar expression used in the workplace prior to the implementation of the EHR system, as an established way of collaborating and solving problems together in everyday work. The concept was thus based on an already existing culture of collaboration that became very important for learning when adapting to new technology. This practice not only addressed shortcomings in formal training but also extended existing ways of collaborating. Participants described that this culture of mutual support made it easier to ask for help and solve problems together when challenges related to the EHR system arose. These brief, task-focused discussions helped to develop shared understandings of how the EHR system works in their real work settings and helped to strengthen competence and individual confidence.

## Discussion

In the following discussion, we consider the study’s findings from a sociocultural perspective. We focus on how learning takes place within communities of practice [38,39] and is shaped by everyday interactions and the organizational context. While learning is mainly social and situated, individual experiences, prior knowledge, and cognitive resources also influence how participants engage with the new EHR system, make sense of it, and move from peripheral to more central participation. By looking at how formal training, peer-based learning, and organizational conditions interact, we aim to understand how healthcare professionals experience and engage in the learning process during EHR implementation.

### Summary of main findings

This study shows how healthcare professionals experienced and engaged in the learning process of implementing a new EHR system. First, the formal training initiatives were perceived as less relevant and poorly adapted to their work practices and digital skills. Second, time limitations and staff availability hindered full participation in learning. Third, peer-based, collective learning forms emerged as a compensatory strategy for inadequate, predefined formal training measures. Overall, this illustrates that learning in EHR implementation is a continuous process related to daily work tasks, social interactions, and organizational frameworks. At the same time, our findings illustrate that learning is also about how health professionals develop as a part of a community of practice [39]. Together, these findings suggest that learning during EHR implementation unfolds through social interaction, participation in everyday work practices, and shared meaning-making among colleagues.

### When training is too far, too soon, and too generic

Within a sociocultural perspective, learning is understood as something that is developed through dialogue, support, and gradual mastery within the individual’s zone of proximal development (ZPD) [36]. By comparison with the theory of ZPD, the training provided three months prior to the roll-out of the new EHR system appeared poorly suited to the actual capabilities of many employees. Both in terms of digital skills and of the transition from a familiar system to a far more complex one, the level expected was far beyond what many were able to build on. As Vygotsky [36] emphasizes, learning activities must take place within a zone where new knowledge can be built upon what one already masters, and where support is provided in a way that enables progression. Within a group with great variations in digital competence, this became especially clear. Although the training allowed for asking questions and being guided by the instructor and peers, it did not sufficiently account for employees with lower levels of digital literacy. This made it hard for them to actively participate in the training.

The training format further exacerbated these challenges. The eight-hour course day spent in front of a computer, detached from everyday working life and without space to gradually build skills, did not provide sufficient support for employees to understand the system’s logic or develop mastery. For several, this made the transition from peripheral participation to more active participation in the simulated practice more challenging than it needed to be. In the absence of real examples from the practice setting or opportunities to test actual workflows, the relevance of the training to everyday practice remained unclear. Such practice-based elements are recognized as crucial for developing understanding and mastery when learning how to use new technology [22,23,28]. Due to this, important forms of practical knowledge, such as who to ask for help, how local adaptations function, and how existing routines can be adjusted, were more difficult to acquire.

The participants’ descriptions of the training as generic and far from their actual practice point to a well-known challenge in simulation. Although aiming to imitate actual practice, studies show that it is challenging to capture the social and unpredictable aspects of clinical practice through simulations [7,8]. Interaction with colleagues, local priorities, time constraints, and continuous trade-offs disappear in a classroom setting, which is precisely the point made by Lave and Wenger [38], where they state that learning is inseparably linked to the social and material contexts in which the work is performed. When training is taken out of this context, important learning conditions are disrupted. Even if the tasks are imitated, learning remains detached from the environment and relationships that give the task meaning. This may also weaken the ability to transfer knowledge to clinical situations. Prevailing studies also reveal that generic training programs for EHR systems do not promote practice-oriented learning, and emphasize that training must be ongoing, anchored in workflow, and flexible so that it can be adapted to different needs and contexts [6,31].

It is also relevant to consider whether the public and professional debates concerning system usability and its alignment with clinical workflows may help explain some of the learning challenges described in this study. Although there are clear pedagogical challenges related to how the training was structured, some of the challenges described here may also reflect the broader demands placed on healthcare professionals as they adapt their practices to a complex EHR system.

Overall, these findings point to two main challenges: (1) the training was outside the development zone of many employees, and (2) it was conducted far from the work context where the knowledge was to be applied. Seen from a sociocultural learning perspective, this means that the training did little to bridge the gap between course content and everyday work. When training neither considers variations in digital competence nor is situated in actual work tasks, it can easily reinforce the feeling of failure. Thus, our findings suggest that informal colleague-based learning may play an important role in supporting competence development during technological implementations in healthcare settings.

### Fragmented participation: organizational barriers to learning in communities of practice

Our findings show that lack of time and staffing, conditions that are well-known challenges in many smaller municipalities [5,6], had a direct impact on employees’ learning opportunities. Despite the training being both planned and structured, it often had to be set aside when clinical tasks demanded attention. This was particularly the case for superusers, who in several instances were unable to participate in meetings regarding the EHR system with other superusers and managers because they needed to cope with tasks that other available employees did not have the authority or competence to take over. Similar challenges are reported internationally and in Nordic studies, where time pressure and organizational priorities are drawn as central barriers to acquiring and developing EHR-related competence [7,25,26].

When superusers lack opportunities to participate in meetings that could increase their EHR expertise, or when other employees are unable to set aside time for practice, this impacts the competence development in the work community. As a sociocultural learning perspective points out, learning occurs through participation, dialogue, and exploration in concrete situations [36]. Time and staffing are, therefore, not only practical frameworks but also decisive for whether such situations may occur at all. Although the participants in the study do not describe explicit shortcomings related to reflection or collective discussions, the time pressure can be understood as something that also limits the scope for such actions.

Superusers play a vital role in this learning process, supporting employees through guidance in learning spaces and *in situ* problem-solving as it arises. Although they are not described as unavailable by the participants, our findings show that superusers were often deprived of opportunities to develop their expertise because they were rarely able to participate in planned professional follow-up activities. When viewed in terms of Vygotsky [36] ZPD, this implies more than just the loss of practical support. Superusers function as ‘more knowledgeable others’ who support employees to move within their ZPD. When their ability to participate in training and professional forums is weakened, their ability to function as what Wood et al. [37] describe as ‘scaffolders’ is weakened. When superusers’ ability to function as scaffolders is reduced, it becomes more challenging for employees to build expertise step by step, and many are left with less support to understand and apply the system fully in their everyday practice. In this sense, superusers’ learning opportunities affect not only their own competence but also that of their colleagues by weakening the framework surrounding the ZPD.

This could also have consequences for the development of competence within the CoP [38,39], as key actors who play a central role in developing common language, routines, and shared understanding lack the capacity to fulfill this role due to time constraints. The introduction of EHR systems introduces new terms, practices, and workflows that employees must learn to navigate. This ‘language’ develops socially through daily use, collective clarification, and joint exploration, forming what Wenger [39] refers to as shared repertoire. When opportunities for such participation are limited, the development of this shared repertoire is weakened. Competence development may become more fragmented, with employees developing different, and sometimes conflicting, ways of understanding [6,27]. When superusers are unable to participate in professional forums or if employees do not have time to observe or solve problems together, the process of developing a shared repertoire and joint understanding risks being weakened. In this way, fragmented knowledge not only becomes an individual challenge but a collective barrier that hinders interaction, knowledge sharing, and good quality in the service.

To build a sustainable community of practice in the face of EHR implementation, individual effort and motivation are not sufficient. This means that organizational frameworks must provide time and space for both formal learning initiatives and collective competence development. Our findings show that this space was limited at the institution studied, which highlights how important it is that training measures and implementation strategies are realistically adapted to the actual resources and working conditions in the municipalities. Training that is tailored to idealized conditions, such as a stable workforce, sufficient time for adequate follow-up, and continuous practicing, risks missing the actual needs in the field.

Our findings show that organizational and structural frameworks, such as sufficient time and staff availability, influence both whether employees can follow structured training plans, but also how learning takes place. When time, staffing, and access to expertise are insufficient, the social and situational learning processes necessary for building competence and establishing new, common practices are weakened. This suggests that facilitating opportunities for shared learning within communities of practice may be important for supporting the implementation of digital systems. How employees attempted to compensate for these challenges through local, informal learning strategies is discussed further in the following section.

### Shoulder-to-shoulder learning as a shared practice and buffer for technological change

The third main finding shows how healthcare professionals actively developed informal, collegial-based learning strategies to cope with the introduction of the EHR system. A central analytical contribution is the concept of ‘shoulder-to-shoulder ‘ learning, which was repeatedly used by the participants and appears as a well-established term within the work community. Participants’ descriptions suggest that the term describes how learning, problem-solving, and collaboration are structured and practiced in daily work. This understanding contributes to making it acceptable, and even expected, to seek help and solve problems together as a response to EHR-related challenges. The shoulder-to-shoulder thus seems to serve as a practical anchor in the community of practice [38], in that short, situated learning sequences provide legitimacy as both meaningful and sufficient.

The meaningful core of the concept is thus expanded to include more than just a description of a collaborative and problem-solving practice, but as a part of the community of practice’s shared repertoire [39]. Implicit norms guide practice, such as the legitimacy of asking for help, the availability of colleagues to provide support, and the collective ownership of expertise. Language thus reflects and shapes practice and guides employees’ responses to EHR-related challenges. Shoulder-to-shoulder learning can thus be understood as an illustration of Wenger’s [39] concepts of mutual engagement and joint enterprise. In these learning situations, employees engage with one another around the EHR system through shared exploration and discussion, while simultaneously adapting work practices in response to system requirements and local clinical needs. The collective goal of adapting the EHR system to their clinical reality constitutes a joint enterprise [39] and is expressed, for example, through adapting workflows, adjusting routines, and performing tasks in accordance with system requirements.

Together, these processes indicate that informal, relational learning may also contribute to sustaining a sense of belonging and shared responsibility within the work community, where engaging in shoulder-to-shoulder learning becomes a way of participating in and contributing to the collective work of the community. In this way, informal learning is closely tied to both the functional and social dimensions of the practice community.

This shoulder-to-shoulder practice also has clear parallels to how learning takes place within the zone of proximal development [36]. When colleagues with more experience or higher digital competence facilitate others in these concrete tasks, they provide situated support that enables them to master tasks they would not have mastered alone. What especially characterizes the findings in this study is that such support is not formally organized or limited to defined superuser-roles, as previous studies often describe [19,20]. Instead, support arises dynamically within the work community, depending on who has relevant competence at any given time. This contributes to a broader distribution of knowledge across the work community and reduces individuals’ vulnerability.

Previous research has shown that collaboration with colleagues can be an effective learning strategy to compensate for gaps in formal training [24,28,30]. Our findings confirm this, but expand the understanding by pointing out that such informal, peer-based processes strengthen the sense of community and shared responsibility for learning. In this way, shoulder-to-shoulder learning functions as more than just a compensatory strategy. Rather, it appears to function as an important practice through which employees support each other when navigating new technological demands in everyday work. At the same time, the effectiveness and sustainability of such practices depend on the conditions that make this type of learning successful: a conscious collaborative culture for learning and the willingness and capacity of individuals to participate in it. This reliance on social dynamics also represents a potential risk of informal learning, as such learning may be difficult to sustain in contexts with high staff turnover.

At the same time, and consistent with previous research, it is important to recognize that informal learning strategies are not exclusively positive. Such strategies can also be ineffective and lead to increased patient risk. According to Hart et al. [34], informal clinical communication can lead to fragmented learning due to a lack of structure, potentially compromising patient risk. Other consequences may be that employees resort to workarounds and improvisations to get around the system, which can lead to errors, knowledge silos, and less efficient system use [33]. It is therefore important that organizations focus on balancing the integration of formal training measures, informal peer-based learning, and technological support to optimize both learning outcomes and patient safety.

### Methodological considerations

We collected the interviews shortly after the implementation of the EHR system, and participants may have had limited experience to draw from. The use of managers as gatekeepers may have led to selection bias, resulting in the recruitment of participants with more positive or negative attitudes toward the implementation [50]. To address these limitations, we sought to include participants of different ages and levels of technological expertise, and a six-hour observation session was conducted to gain contextual insight into everyday work practices related to the use of the EHR system.

In qualitative studies, sample adequacy is evaluated based on whether the data material has sufficient information power [51]. According to this concept, the informative value of a study is shaped by factors such as the study aim, the specificity of the participant group, the use of theory, the quality of dialogue during data collection, and the chosen analytical approach. In this study, the sample holds information power because the aim was limited to examining how healthcare professionals experience and engage in training and learning processes during the implementation of an EHR system in a municipal healthcare setting. Furthermore, the participant group was specific, consisting of employees working closely with the implementation in practice. The interviews provided space for explorative dialogue about the participants’ experiences, and the analysis was conducted as a reflective thematic analysis. The study’s information power is further strengthened by the sociocultural perspective on learning, which informed the analytical lens and guided the interpretation of participants’ experiences.

The study is conducted within one municipal healthcare institution and involves a specific group of healthcare professionals. The findings therefore must be understood in relation to this context, and their relevance to other contexts should be evaluated in light of comparable organizational and professional conditions.

### Conclusion

Overall, the study shows that healthcare professionals experienced the implementation of the EHR system as a learning process that unfolded primarily through everyday work collaboration and peer support, rather than through predefined training programs. Formal training provided an introduction to the system, but it was too decontextualized from real everyday practice to serve as support when the system was introduced. Instead, much of the actual learning took place through informal arenas, through collaboration with colleagues, and through shared problem-solving in daily work.

Instead, professionals engaged in learning through informal, practice-based arenas, working shoulder-to-shoulder, troubleshooting together, and gradually developing shared ways of understanding and managing the new system. Over time, this practice has led to a shared understanding of how learning takes place in the workplace, not only in terms of how to improve technological competence, but also as part of belonging to and contributing to a professional community.

At the same time, the findings highlight how organizational conditions shape these learning processes. Limited time, staffing constraints, and few formal arenas for shared reflection can limit opportunities for collective learning. Nevertheless, the study shows how healthcare professionals used the resources they had, their relationship with colleagues, routines, and mutual trust and support, to create situated solutions and build competence regarding the EHR together.

### Implications for practice

The findings of this study highlight the need for training programs that combine courses with more experience-based learning approaches. For this to be effective in practice, those designing the training need to consider the limited resources available in smaller municipalities and adapt the training accordingly. Although managers may recognize the value of practice-based learning in CoPs, it is more challenging to implement this form of learning when the necessary resources are lacking. In healthcare institutions with limited staffing levels, particularly in smaller municipalities, it becomes even more crucial to support such communities, as they can help compensate for inadequate formal training in implementing EHRs. This may involve ensuring that superusers have time to participate in training and professional meetings, providing structured opportunities for in-situ support for employees in the early stages of system use, and adapting training activities to different levels of digital competence. At the same time, organizations should ensure that informal peer-based learning is supported by appropriate structures and guidance in order to reduce the risk of fragmented learning and unsafe workarounds.

### Proposed areas for future research

Future studies should examine how communities of practice evolve over time due to technological change, focusing especially on peer-based learning strategies, such as shoulder-to-shoulder practices. By examining how such practices emerge over time and how organizational conditions affect this, one could reveal what supports both individual and collective competence development in the face of technological change. These insights would be especially valuable for institutions where structures limit opportunities for formal training and where competence development to a larger extent depends on socially embedded practices within the work community. Future studies could also explore how organizations balance formal training initiatives, informal peer-based learning, and technological support in order to support safe and effective competence development during large-scale digital transformations.

## Data Availability

The data supporting the findings of this study are not publicly available due to ethical and privacy considerations but may be made available upon reasonable request.
